# Supplementary material for: Three-dimensional heads-up surgery in ab-interno trabeculotomy: Image processing-assisted trabeculotomy
Source: PLoS One. 2022 Feb 10;17(2):e0263588. doi: 10.1371/journal.pone.0263588 (PMC8830679; doi:10.1371/journal.pone.0263588)
Supplement: Attachment — Submitted filename: Response_to_Reviewers.docx [file pone.0263588.s002.docx]

Emily Chenette

Editor-in-Chief

*PLOS One*

Dear Dr. Chenette:

We would like to re-submit the attached manuscript entitled “**Three-dimensional heads-up surgery in ab-interno trabeculotomy: Image processing assisted trabeculotomy**.” The manuscript ID is PONE-D-21-18797.

The manuscript has been carefully rechecked and appropriate changes have been made in accordance with the reviewers’ suggestions. The responses to their comments have been prepared and attached herewith.

We thank you and the reviewers for the thoughtful suggestions and insights, which have enriched the manuscript and produced a more balanced and better account of the research. We hope that the revised manuscript is now suitable for publication in your journal.

We look forward to your reply.

Sincerely,

Takashi Fujishiro MD, PhD

Department of Ophthalmology,

University of Tokyo Graduate School of Medicine

**<Answers to the editor and the reviewer’s comments>**

We wish to express our appreciation to the reviewer for the insightful comments. The revisions in the manuscript have been marked using yellow marker, and our point-by-point responses to the reviewer’s comments are provided below.

**Journal Requirements:**

**1. Please ensure that your manuscript meets PLOS ONE's style requirements, including those for file naming. The PLOS ONE style templates can be found at**

[**https://journals.plos.org/plosone/s/file?id=wjVg/PLOSOne_formatting_sample_main_body.pdf**](https://journals.plos.org/plosone/s/file?id=wjVg/PLOSOne_formatting_sample_main_body.pdf)

**and** [**https://journals.plos.org/plosone/s/file?id=ba62/PLOSOne_formatting_sample_title_authors_affiliations.pdf**](https://journals.plos.org/plosone/s/file?id=ba62/PLOSOne_formatting_sample_title_authors_affiliations.pdf)**.**

Response:

Thank you for the comment. We have revised the the manuscript and file naming to meet PLOS ONE’s style.

**2. Please clarify the nature of the written informed consent in your Ethics Statement and Methods section. Did patients consent to the medical treatment, and/or did they specifically consent to participate in this study, including the use of their medical data for research.**

Response:

Thank you for the comment. The patients agreed to receive the medical treatment and to have treatment data used for research. We have added the following in the Methods section: “(the medical treatment itself and the use of their medical data for research)” (page 10, line 131–132).

**3. We note that you have indicated that data from this study are available upon request. PLOS only allows data to be available upon request if there are legal or ethical restrictions on sharing data publicly. For more information on unacceptable data access restrictions, please see** [**http://journals.plos.org/plosone/s/data-availability#loc-unacceptable-data-access-restrictions**](http://journals.plos.org/plosone/s/data-availability#loc-unacceptable-data-access-restrictions)**.**

**In your revised cover letter, please address the following prompts:**

**a) If there are ethical or legal restrictions on sharing a de-identified data set, please explain them in detail (e.g., data contain potentially sensitive information, data are owned by a third-party organization, etc.) and who has imposed them (e.g., an ethics committee). Please also provide contact information for a data access committee, ethics committee, or other institutional body to which data requests may be sent.**

**b) If there are no restrictions, please upload the minimal anonymized data set necessary to replicate your study findings as either Supporting Information files or to a stable, public repository and provide us with the relevant URLs, DOIs, or accession numbers. For a list of acceptable repositories, please see http://journals.plos.org/plosone/s/data-availability#loc-recommended-repositories.**

Response:

Thank you for the comment. Our datasets do not have any restrictions. Thus, we have uploaded the minimal anonymized dataset such as the measured values of the luminance in pig eyes and human eyes, and the working distance in Excel format as Supporting Information files.

**Reviewer #1:**

**1. In page 12, the experimental conditions explained for both pig and human eyes are different. Could you please explain the difference in testing conditions if the new model of surgery has to be implemented then testing conditions need to be same for all practical purposes.**

Response:

Thank you for the comment. We apologize for misleading you about the main outcome.

The main outcome of the current study was not to find a universal optimal image condition, but to show whether image processing can improve visibility.

As you pointed out, in order to find the optimal image condition, it is necessary to use the same imaging condition for both human eyes and pig eyes. However, for safety in the human surgery, the surgeon had to perform the TM incision under the conditions that he felt were easiest for looking at the TM (only contrast-enhanced condition 1’, which was different from condition 1 used in pig eyes). For comparison, we further increased the contrast and took images in condition 2’. We then examined whether the difference in luminance, which is an indicator of visibility, improved under these three types of conditions.

We have deleted the sentence “In the future, we hope to develop optimal microscope image settings for various situations” at the end of the Discussion section.

We added the following limitation in the Discussion section: “Second, in order to find the optimal imaging condition, it is necessary to use the same imaging condition for both human eyes and pig eyes. However, for safety in the human surgery, the surgeon had to perform the TM incision under the conditions that he felt were easiest for looking at the TM (only contrast-enhanced condition 1’, which was different from condition 1 used in pig eyes)” (page 22, line 334–338).

**2. Table 2 results – in Human eyes the mean and standard deviation provided in table appears to be statistically not viable as standard deviation is more than or equal to 1/3 rd of mean. Eg The differences in luminance (mean values and standard deviations) were 21.1 ± 7.3 arb. units without processing', 28.1 ± 12.1 arb. units under condition 1', and 17.5 ± 15.6 arb. units under condition 2'.**

Response:

Thank you for the comment. As you pointed out, we think the parametric test was not suitable because of the high variability of the data in human eyes. Therefore, we increased the number of cases from 8 cases to 11 cases to reduce the variability and then performed a non-parametric test. Just like before, the results showed that there was a significant difference between No processing’ and Condition 1', and no significant difference between No processing’ and Condition 2'. We have also changed the notation to be median and interquartile range instead of mean ± standard deviation. To reflect the previous description, we have changed the number of cases from 8 to 11 in the corresponding parts of the main manuscript. In addition, we have changed the contents of Table 2 (page 15) and Figure 5, and added a sentence in the Methods section as follows: “In contrast, the median value and interquartile range for each imaging condition were calculated and compared using Wilcoxon signed-rank test, with a significance level of p < 0.05 because of the variation of the measured values in the human eyes” (page 15, lines 209–212). We have also changed the values in the Results section as follows: “The median (interquartile range) differences in luminance were 18.8 (15.8–21.2) arb. units without processing, 21.8 (21.0–27.4) arb. units under condition 1', and 8.5 (4.3–14.7) arb. units under condition 2'” (page 16, lines 235–239).

**3. Has the data been checked for normality before applying paired t test –seems inappropriate as the standard deviation is > 1/3 of mean. A non-parametric test would have been appropriate.**

Response:

Thank you for the suggestion. As you pointed out, in human eyes, we agree that nonparametric tests are suitable because of the variation of the measured values. Therefore, the Wilcoxon signed-rank test was performed after we increased the number of cases. We added the following information in the Methods section: “In contrast, the median value and interquartile range for each imaging condition were calculated and compared using the Wilcoxon signed-rank test, with a significance level of p < 0.05 because of the variation of the measured values in the human eyes” (page 15, lines 209–212).

**4. Swan Jacob lens spelling incorrectly spelt in page 9 line 123.**

Thank you for the comment. We have revised the spelling.

**5. corner angle should have been corneal angle in page 19 line 282.**

Thank you for the comment. We have revised the spelling.
